# Pituitary adenylate cyclase-activating polypeptide modulates circadian feeding and emotional behavior in mice independent of stress-induced hyperphagia

**DOI:** 10.14202/vetworld.2025.3218-3228

**Published:** 2025-10-31

**Authors:** Ha Thi Thanh Nguyen, Thi Ngan Mai, Thi Thu Tra Vu, Thanh Trung Nguyen

**Affiliations:** 1 Department of Veterinary Pharmacology and Toxicology, Faculty of Veterinary Medicine, Vietnam National University of Agriculture, Gia Lam District, Hanoi, Vietnam; 2 Laboratory of Pharmacology and Drug Development, Center of Excellence Research and Innovation, Vietnam National University of Agriculture, Gia Lam District, Hanoi, Vietnam; 3 Department of Veterinary Microbiology and Infectious Diseases, Faculty of Veterinary Medicine, Vietnam National University of Agriculture, Hanoi, Vietnam; 4 Department of Veterinary Public Health, Faculty of Veterinary Medicine, Vietnam National University of Agriculture, Hanoi, Vietnam

**Keywords:** circadian feeding, emotional behavior, hyperphagia, pituitary adenylate cyclase-activating polypeptide, stress, ventromedial hypothalamus

## Abstract

**Background and Aim:**

Pituitary adenylate cyclase-activating polypeptide (PACAP) is a neuropeptide widely implicated in stress responses, appetite regulation, and emotional behavior. While PACAP has been linked to stress-induced appetite suppression, its role in circadian feeding and stress-related hyperphagia remains poorly defined. This study aimed to clarify the contribution of PACAP to circadian feeding behavior, chronic stress-induced hyperphagia, and emotional regulation in mice.

**Materials and Methods:**

Experiments were conducted using PACAP knockout (KO) and wild-type (WT) CD-1 mice. Acute and chronic restraint stress paradigms were applied, and food intake, body weight, and behavioral assays were recorded. Viral-mediated PACAP overexpression in the ventromedial hypothalamus (VMH) was performed using adeno-associated vectors. Emotional regulation was assessed through forced swim and tail suspension tests (TSTs). PAC1 receptor expression was quantified by reverse transcription and quantitative polymerase chain reaction.

**Results:**

PACAP overexpression in the VMH significantly increased nocturnal feeding, demonstrating a circadian-specific effect on appetite regulation. Chronic restraint stress enhanced food intake in both PACAP KO and WT mice, whereas acute stress showed no effect, indicating that chronic stress-induced hyperphagia occurs independently of PACAP signaling. Behaviorally, PACAP-overexpressing mice exhibited reduced immobility in forced swim and TSTs, consistent with enhanced stress resilience and antidepressant-like effects. Importantly, PAC1 receptor expression remained stable throughout the diurnal cycle, suggesting that PACAP’s modulatory effects are driven by neuropeptide availability rather than receptor fluctuations.

**Conclusion:**

This study identifies PACAP in the VMH as a key modulator of circadian feeding and emotional behavior, while demonstrating its non-essential role in chronic stress-induced hyperphagia. The findings suggest that PACAP selectively integrates circadian and emotional signals to regulate feeding, independent of compensatory neuropeptide systems that mediate stress hyperphagia. These insights advance the understanding of neuropeptide regulation of energy balance and mood, with implications for stress-related eating disorders and anxiety.

## INTRODUCTION

Pituitary adenylate cyclase-activating polypeptide (PACAP) is a member of the vasoactive intestinal peptide (VIP)/secretin/glucagon/growth hormone-releasing hormone neuropeptide family. It was first discovered in the ovine hypothalamus due to its potent ability to stimulate cyclic adenosine monophosphate production in rat pituitary cells [1]. PACAP exerts its effects through three G-protein-coupled receptors: PAC1, which binds PACAP38 and PACAP27 with high affinity and preferentially activates the adenylate cyclase/PKA pathway, and VPAC1 and VPAC2, which recognize both PACAP and VIP with similar affinity and primarily couple to adenylate cyclase [2, 3].

Functionally, PACAP plays a central and multifaceted role in regulating physiological and behavioral stress responses in mice [4–6]. It is abundantly expressed in stress-sensitive brain regions, including the hypothalamus, amygdala, and brainstem [7]. Through these regions, PACAP stimulates the hypothalamic–pituitary–adrenal (HPA) axis by promoting the release of corticotropin-releasing hormone (CRH), which subsequently triggers adrenocorticotropic hormone (ACTH) and corticosterone secretion [8]. In addition, PACAP enhances sympathetic nervous system activity by facilitating adrenal catecholamine release [6, 9].

Beyond its neuroendocrine actions, PACAP strongly influences stress-related behaviors such as anxiety and depression [10, 11]. Mice lacking PACAP (PACAP-knockout [KO]) display blunted hormonal stress responses and altered coping behaviors [12], underscoring its essential role in stress adaptation. Consequently, PACAP has been proposed as a promising therapeutic target for stress-associated disorders, including anxiety, post-traumatic stress disorder, and depression, through its modulation of the HPA axis, autonomic function, and emotional neural circuits [10, 13, 14].

In addition to stress regulation, PACAP contributes to appetite control, particularly under stressful conditions [15–18]. Within the hypothalamus, it interacts with appetite-regulating neurons that produce neuropeptides such as neuropeptide Y (NPY), agouti-related peptide, and proopiomelanocortin [17, 19, 20]. Typically, food intake is suppressed during stress, and PACAP is believed to mediate this anorexigenic response [21]. Importantly, PACAP-KO mice often fail to exhibit stress-induced reductions in food intake [22], suggesting that PACAP is a key mediator of appetite suppression under stress.

Although PACAP is well recognized as a neuropeptide orchestrating stress responses and influencing feeding behavior, several aspects of its role remain unresolved. Existing studies have largely emphasized PACAP’s involvement in activating the HPA axis and mediating acute stress-induced anorexia, but its contribution to long-term adaptations under chronic stress conditions is not clearly defined. In particular, whether PACAP is indispensable for stress-induced hyperphagia remains controversial, as KO models sometimes display paradoxical increases in food intake rather than suppression. Similarly, while PACAP has been shown to interact with circadian pathways and influence photic entrainment of biological rhythms, its specific involvement in regulating circadian feeding patterns has not been systematically addressed. Another gap concerns the interaction between PACAP and emotional regulation in relation to feeding: although PACAP has documented anxiogenic effects through limbic circuits, the extent to which these emotional influences overlap with metabolic control is poorly understood. Furthermore, the stability of PAC1 receptor expression across circadian phases raises the question of whether PACAP’s effects are driven primarily by temporal fluctuations in peptide availability rather than receptor dynamics. Together, these gaps hinder a comprehensive understanding of how PACAP integrates stress, circadian rhythms, and emotional behavior to regulate appetite and energy balance.

The present study aimed to dissect the specific role of PACAP in regulating feeding behavior under both stress and non-stress conditions, with a focus on circadian and emotional influences. Using PACAP KO and wild-type (WT) mice, as well as viral-mediated PACAP overexpression in the ventromedial hypothalamus (VMH), we sought to determine: (i) whether PACAP is required for the development of stress-induced hyperphagia under acute and chronic restraint stress; (ii) whether PACAP modulates circadian feeding, particularly during the nocturnal phase; and (iii) how PACAP overexpression influences emotional responses in stress-related behavioral paradigms. By clarifying these roles, the study aims to distinguish between essential and modulatory functions of PACAP, thereby advancing our understanding of neuropeptide regulation of appetite and stress-related behaviors. These insights are expected to provide a mechanistic basis for targeting PACAP signaling in the context of eating disorders, obesity, and mood-related pathologies.

## MATERIALS AND METHODS

### Ethical approval

The experimental animal research committees of Kagoshima University (Approval No. MD18105) and Vietnam National University of Agriculture (Approval No. CARE-2023/05) approved all animal procedures. Animals were housed individually in standard polycarbonate cages under a controlled temperature (22°C ± 2°C), 50%–60% humidity, and a 12-h light/dark cycle (lights on at 7:00 AM). Mice were randomly allocated to groups. All procedures adhered to the institutional guidelines and the Animals in Research: Reporting *In Vivo* Experiments 2.0 reporting standards, and the National Institutes of Health Guide for the Care and Use of Laboratory Animals. PACAP-KO mice were previously developed and backcrossed onto a CD-1 genetic background [23]. All experiments were conducted using male CD-1 mice (Japan SLC Inc., Shizuoka, Japan) aged between 8 and 13 weeks. Mice were housed individually under a controlled 12-h light/dark cycle (lights on at 7:00 A.M. and off at 7:00 P.M.) for at least 1 week before and throughout the experimental procedures.

### Study period and location

The study was conducted at Kagoshima University from January 2018 to December 2019 and at the Vietnam National University of Agriculture from January 2023 to December 2024.

### PACAP genotyping

The method for genotyping was adapted from a previously published protocol by Nguyen *et al*. [17] and Hashimoto *et al*. [23], and >10 generations were backcrossed onto a CD-1 background. Ear punch samples (0.25 cm) were collected from mice and incubated for 1 h in 50 mM NaOH at 95°C. After incubation, the samples were neutralized with 1 M Tris-HCl (pH 8.0). Genotyping was conducted through polymerase chain reaction (PCR) using the primers listed in Table 1 under the following cycling conditions: Initial denaturation at 95°C for 3 min, followed by 40 cycles of denaturation at 95°C for 30 s, annealing at 59°C for 30 s, and extension at 72°C for 2 min. The final step was a hold at 4°C. The PCR products were then separated and visualized on 1% agarose gels.

**Table 1:** Primer sequences used for genotyping.

Targets	Expected size (bp)	Forward (5’- 3’)	Reverse (5’- 3’)
PACAP (+/+)	293	ACCGAAAACAAATGGCTGTC	GGTCCACAAAGTATATCTGTGCATTCTC
PACAP (-/-)	1078	ATCTCCTGTCATCTCACCTTGCTCCT	GGTCCACAAAGTATATCTGTGCATTCTC

PACAP = Pituitary adenylate cyclase-activating polypeptide

### Acute and chronic restraint stress strategies

The ARS procedure was adapted with minor modifications from a previously published method by Huang *et al*. [24]. For acute stress, mice were restrained for 15 min in ventilated 50 mL tubes on a single occasion. For chronic stress, this procedure was repeated 15 min/day for 3 consecutive days. Food intake and body weight were monitored at consistent time points before and after the restraint periods. Body weight and food intake were assessed at 10 AM before and after acute stress exposure and on days 1, 2, and 3 during chronic stress. Upon completion of the final assessment, the mice were euthanized by cervical dislocation.

### Construction and preparation of the adeno-associated virus (AAV) vector

The virus was prepared with the aforementioned slight modifications [25]. The coding region of mouse PACAP was amplified using primers 5′-ATGACCATGTGTAGCGGAGC-3′ and 5′-CTACAAGTATGCTATTCGGCGTC-3′, corresponding to the sequence annotated under GenBank accession number NM_009625.5, and then cloned into the pAAV-CAG::IRES-EGFP plasmid to generate pAAV-CAG::PACAP-IRES-EGFP. AAV5 vectors were produced using the AAVPro Helper Free System (Takara, Shiga, Japan). Equal amounts of pAAV, pRC5, and pHelper plasmids were co-transfected into HEK293T cells using 1 mg/mL polyethyleneimine (PEI) (Polyscience, Germany). After 3 days, the cells were harvested and lysed in phosphate-buffered saline (pH 7.4) by freeze-thaw cycles. The lysates were treated with benzonase (Merck Millipore, Germany) and purified using OptiPrep gradient ultracentrifugation (Abbott, Norway). The resulting AAV virions were concentrated using Vivaspin 20 (Sartorius, Japan), and their titers were quantified as described by Aurnhammer *et al*. [26]. Aliquots were stored at – 80°C until use.

### Stereotaxic surgery and viral injection (VI)

Stereotaxic surgery was performed as described by Krashes *et al*. [27]. Mice were anesthetized by intraperitoneal injection of a mixture containing medetomidine (0.75 mg/kg), midazolam (4.0 mg/kg), and butorphanol (5.0 mg/kg) [28]. After drilling cranial holes, 32G stainless steel needles were stereotaxically inserted, and AAVs (2 × 10^13^ vg/mL) were injected bilaterally at a rate of 0.1 μL/min for 10 min. The needles were left in place for an additional 5 min to allow viral diffusion before removal. Coordinates were determined according to Paxinos and Franklin’s mouse brain atlas: anterior–posterior, 1.4 mm; medial–lateral, ±0.4 mm; dorsal–ventral, 4.8 mm. Site accuracy was confirmed postmortem through green fluorescent protein fluorescence microscopy [29]. Following suturing, atipamezole (0.75 mg/kg) and penicillin G (0.8 mg/mouse) were administered to induce recovery and prevent infection, respectively. Animals with surgical failure or injection misplacement will be excluded from the analysis if applicable. Behavioral testing, body weight, and food intake were evaluated 3 weeks after the injection.

### Cell culture and gene transfection

HEK293T cells (passages 5–15) were cultured in Dulbecco’s Modified Eagle Medium (Gibco, MA, USA) supplemented with 10% fetal bovine serum (Gibco) and 1% penicillin-streptomycin. Cells were maintained at 37°C in a humidified incubator with 5% CO_2_. When the cultures reached approximately 90% confluency, the cells were transiently transfected with the pAAV-CAG::PACAP-IRES-EGFP plasmid using PEI (Polysciences, PA, USA) according to the manufacturer’s protocol [17]. Total RNA was extracted after 48 h, and immunocytochemistry was conducted.

### Forced swim test (FST)

The FST, a well-established measure of behavioral despair in mice [30, 31], was conducted using glass cylinders (20 cm height and 10 cm diameter) filled with water to a depth of 10 cm at 25°C. On the 1^st^ day, mice were individually placed in the cylinders for 5 min, then removed, dried, and returned to their home cages. Twenty-four hours later, the mice were re-exposed to the same conditions. Following a 1-min acclimation period, the immobility was recorded for 5 min. Mice were considered immobile when they remained passively floating without struggling and were completely motionless. Testing was conducted between 9:00 AM and 12:00 PM during the light phase. Mice were allowed to inhabit the testing room for 1 h before testing. All animals were randomly assigned to groups, and an experimenter blinded to treatment performed behavioral scoring. The cylinders were cleaned and refilled between trials.

### Tail suspension test (TST)

The TST was conducted as mentioned earlier [31, 32]. Mice were individually suspended by the tail using adhesive tape placed 1 cm from the tip and hung 60 cm above the table surface in a designated TST box. After a 1-min acclimation period, the immobility was recorded for 5 min. Mice were considered immobile when they hung passively and remained motionless. All tests were conducted during the light phase between 9:00 AM and 12:00 PM. The mice were habituated to the testing room for at least 1 h before testing. Animals were randomly assigned to groups using a computerized algorithm, and an experimenter blinded to group allocation performed behavioral scoring.

### Reverse transcription and quantitative PCR (RT-qPCR)

Total RNA was extracted from the hypothalamus region using the Sepasol-RNA 1 Super G kit (Nacalai Tesque, Kyoto, Japan) according to the manufacturer’s instructions. Complementary DNA (cDNA) was synthesized from the extracted RNA using the high-capacity cDNA reverse transcription kit (Applied Biosystems, Foster City, CA, USA). qPCR was performed using the Thunderbird SYBR qPCR kit (Toyobo Life Science, Osaka, Japan) on a Thermal Cycler Dice Real-Time System TP800 (Takara Bio Inc., Shiga, Japan). The specific primer sequences used for PAC1R were: forward 5′-GTGCAGTACGCACACACCG-3′ and reverse 5′-AGCCTTGGGGAGTCAGTCAC-3′. For glyceraldehyde-3-phosphate dehydrogenase (GAPDH), the internal control, the primers were: forward 5′-GAAGGTCGGTGTGAACGGAT-3′ and reverse 5′-CTCGCTCCTGGAAGATGGTG-3′. PAC1R expression levels were normalized to GAPDH and reported as relative expression units. The amplification efficiency ranged from 95% to 102%, and the specificity was confirmed via single-peak melt curve analysis. The stability of the reference gene (GAPDH) was validated using the geNorm method.

### Statistical analysis

Results are presented as means ± standard error of the mean. Statistical significance was assessed using Student’s t-test for pairwise comparisons or analysis of variance for genotype × treatment effects, followed by Dunnett’s *post hoc* test, performed using Prism 4.3 software (GraphPad, San Diego, CA, USA). p = 0.05 was considered statistically significant. The Shapiro–Wilk test was used to assess data normality. Any missing data were excluded from the analyses and excluded from the experimental results.

## RESULTS

### PACAP promotes nocturnal feeding

**Figure 1:**
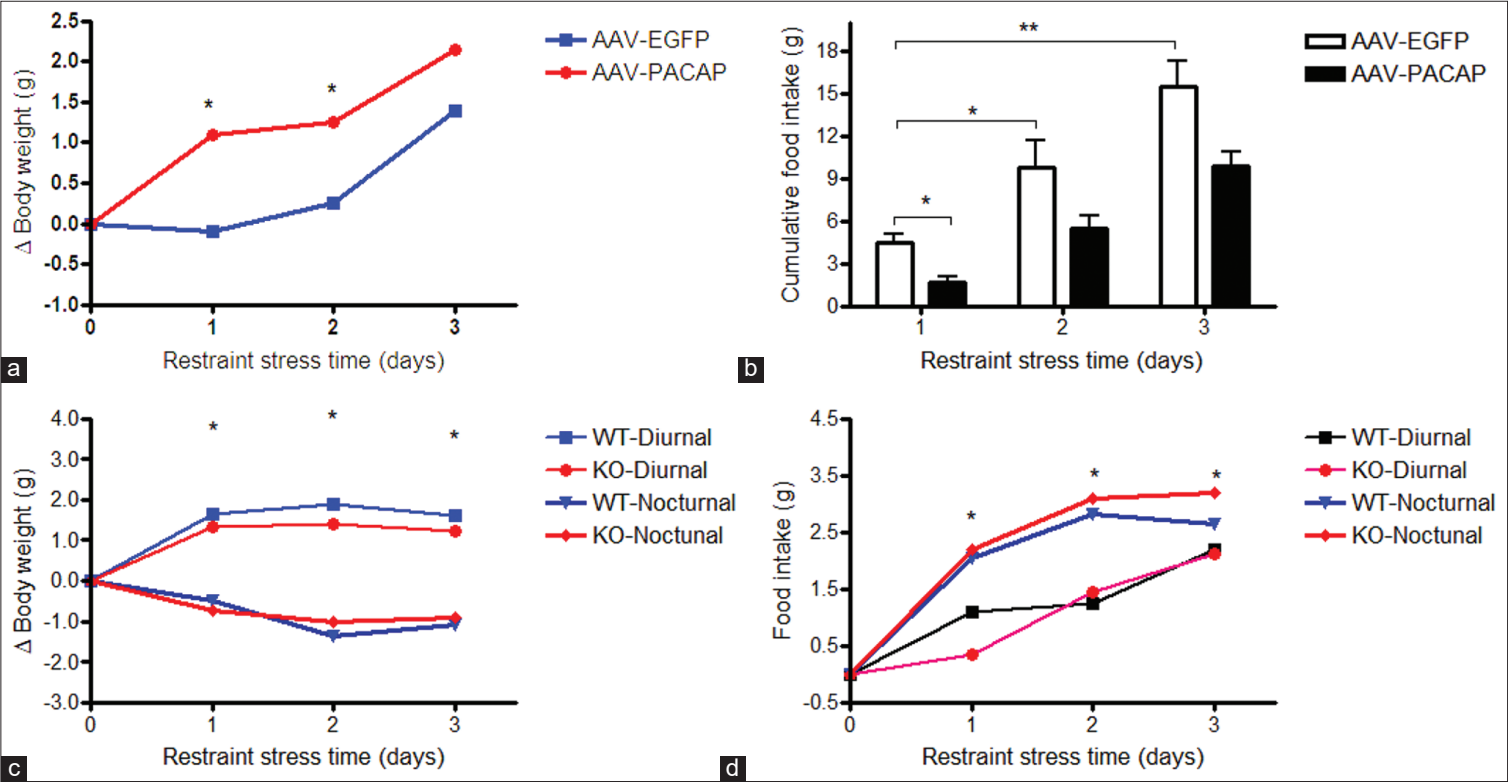
PACAP promotes nocturnal feeding. (a and b) Body weight gain and food intake following PACAP overexpression in the VMH during 1–3 days of restraint stress. (c and d) Body weight gain and cumulative food intake in PACAP wild-type (+/+) and knockout mice subjected to 1–3 days of restraint stress. Data are presented as the mean ± Standard Error of the Mean (two-way analysis of variance, *post hoc* Dunnett test, n = 4 mice per group). *p < 0.05, **p < 0.01. PACAP = Pituitary adenylate cyclase-activating polypeptide, VMH = Ventromedial hypothalamus.

As shown in Figure 1, PACAP overexpression in the VMH significantly increased food intake during the nocturnal phase in WT mice. This finding suggests that PACAP promotes feeding behavior specifically during the dark period, which is consistent with its role in regulating energy balance and circadian rhythms. These results suggest that PACAP may integrate circadian signals with metabolic needs to modulate feeding under normal physiological conditions.

### Chronic stress-enhanced feeding independent of PACAP

**Figure 2:**
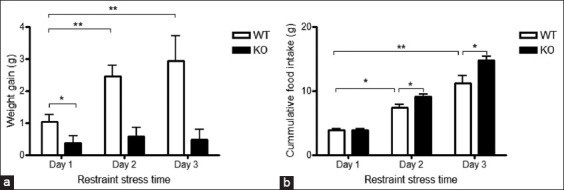
Chronic stress-induced feeding behavior occurs independently of the PACAP group. (a) Body weight gain and (b) cumulative food intake in PACAP wild-type (+/+) and knockout mice subjected to restraint stress for 1–3 days. Data are presented as mean ± Standard Error of the Mean (two-way analysis of variance, *post hoc* Dunnett test, n = 6 mice per group). *p < 0.05; **p < 0.01. PACAP = Pituitary adenylate cyclase-activating polypeptide.

As illustrated in Figure 2, chronic restraint stress led to a significant increase in food intake in both PACAP KO and WT mice, whereas acute stress exposure did not affect feeding behavior in either group. These findings suggest that the hyperphagic response to prolonged stress is independent of PACAP signaling, indicating that other neurobiological mechanisms may compensate for the absence of PACAP during chronic stress.

### PACAP enhances stress-coping behavior

**Figure 3:**
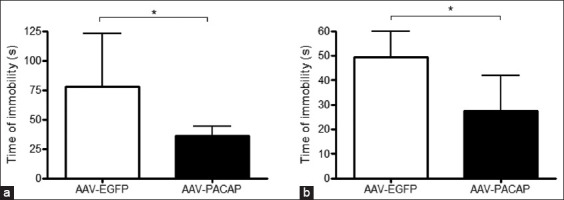
Reduced immobility in PACAP-overexpressing mice indicates antidepressant-like activity. (a and b) Immobility time in the FST and TST following PACAP overexpression in the VMH region. Data are presented as mean ± standard error of the mean (Student’s t-test for pairwise comparisons, n = 3 mice per group). *p < 0.05, Student’s t-test. PACAP = Pituitary adenylate cyclase-activating polypeptide, VMH = Ventromedial hypothalamus, FST = Forced swim test, TST = Tail suspension test.

To evaluate the impact of PACAP overexpression on depression-like behaviors, the FST and TST were conducted. As shown in Figure 3, PACAP-overexpressing mice exhibited a significant decrease in immobility time in both tests compared with controls. This reduction in passive behavior suggests enhanced stress resilience and antidepressant-like effects. These findings support the functional role of PACAP in modulating mood-related responses under stress.

### Stable PAC1 receptor expression across the day

**Figure 4:**
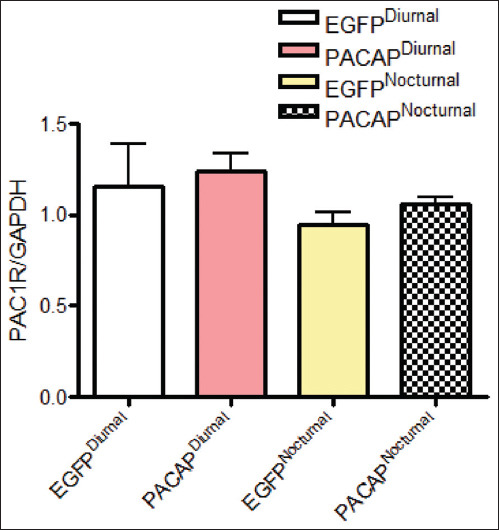
PAC1 receptor expression remains stable. PAC1R mRNA expression levels in the VMH region during diurnal and nocturnal periods following selective PACAP overexpression, as measured by RT-qPCR (two-way analysis of variance, *post hoc* Dunnett test, n = 3 mice per period). PACAP = Pituitary adenylate cyclase-activating polypeptide, VMH = Ventromedial hypothalamus, mRNA = Messenger RNA, RT-qPCR = Reverse transcription and quantitative polymerase chain reaction.

As shown in Figure 4, the PAC1 receptor expression remained stable throughout the day. This suggests that the effects of PACAP are unlikely to be driven by fluctuations in receptor levels, but rather by changes in PACAP availability or release, which may depend on circadian rhythms or physiological contexts, such as stress or metabolic state. Consistent receptor expression provides a stable platform for dynamic PACAP signaling.

## DISCUSSION

### Differentiating PACAP functions in feeding behavior

To the best of our knowledge, this is one of the first studies to differentiate the role of PACAP in chronic stress-induced feeding from its role in circadian nocturnal feeding, highlighting the critical versus non-essential aspects of each. Furthermore, PACAP overexpression in the VMH links emotional regulation with feeding behavior, thereby bridging the domains of circadian and stress regulation. These findings contribute to the growing body of evidence that PACAP influences energy balance and behavior in a complex and context-dependent manner.

### PACAP and stress-induced hyperphagia

We found that chronic restraint stress increased food intake in both PACAP-KO and WT mice, whereas acute stress had no effect in either group, suggesting that stress-induced hyperphagia during chronic exposure is independent of PACAP signaling. Although PACAP is involved in the hypothalamic stress axis and mediates anorexic responses during acute stress, its absence does not impair long-term behavioral adaptation to repeated stress. This may reflect the compensatory actions of other neuropeptides, such as NPY, CRH, or ghrelin, suggesting that these compensatory neuropeptides may replace PACAP during chronic stress. While PACAP regulates HPA axis activity, it is not essential for glucocorticoid release induced by CRH or ACTH [33, 34]. PACAP appears to act upstream, at or above the hypothalamus, to mediate HPA axis activation in response to psychogenic, but not physiological, stress. Indeed, PACAP-KO mice show blunted corticosterone responses to acute and chronic psychogenic stressors (e.g., restraint, social defeat, open field) but normal responses to physiological challenges (e.g., sepsis, hypoglycemia, pain, cold, ether) [9, 33], highlighting the selective role of PACAP in central nervous system-mediated stress regulation.

### Psychological and environmental influences

Interestingly, emotional state and dietary context may also modulate stress-induced changes in feeding behavior. Studies by Markus *et al*. [35], Barr *et al*. [36], and Utter *et al*. [37] have suggested that stress relief is associated with increased carbohydrate intake and food choice, whereas stress promotion, characterized by limited dietary options and persistent negative affect, is linked to reduced carbohydrate intake and increased adiposity. These findings imply that both psychological and environmental factors influence feeding responses under stress, possibly interacting with neuropeptide systems in a complex, context-dependent manner. Future research should aim to identify specific neuropeptide systems that compensate for the absence of PACAP during chronic stress-induced hyperphagia. Investigating the roles of NPY, CRH, and ghrelin in PACAP-KO models under chronic stress conditions may help clarify the mechanisms underlying PACAP-independent adaptation. In addition, assessing region-specific PACAP or PAC1R manipulations in brain areas such as the bed nucleus of the stria terminalis (BNST), hypothalamus, and amygdala to determine their contributions to stress-induced feeding behavior would be valuable.

### PACAP and circadian modulation of feeding

Interestingly, PACAP overexpression in the VMH of WT mice led to increased food intake during the nocturnal period, suggesting that PACAP contributes to the modulation of feeding circadian rhythms. Moreover, our previous study by Nguyen *et al*. [17] indicated that PACAP modulates feeding in a phase-dependent manner, which is consistent with the literature showing opposing roles of PACAP (e.g., anorexigenic versus orexigenic effects) across the light–dark cycle. These findings support the view that PACAP acts as a context-dependent modulator, rather than a universal regulator, of stress-induced feeding. This finding aligns with previous studies by Hannibal [38], Damato and Herzog [39], and Naveed *et al*. [40], which show that PACAP plays a role in the photic entrainment of circadian rhythms, supporting the notion that PACAP acts in synchrony with the central circadian clock to modulate behavior and metabolism. The selective effect during the dark phase implies that PACAP may enhance feeding drive when metabolic demands are naturally elevated.

### PACAP and emotional regulation of feeding

Our findings also demonstrate that PACAP overexpression in the VMH increases anxiety-like behavior, which aligns with earlier studies by Maita *et al*. [41] indicating that PACAP exerts anxiogenic effects through limbic structures such as the amygdala and BNST. The anxiogenic profile of PACAP overexpression in VMH may contribute to altered feeding behavior, given the critical role of VMH in satiety control and stress-induced appetite regulation. While heightened anxiety is typically linked to reduced food intake, our findings indicate that PACAP overexpression under chronic stress conditions may instead lead to dysregulated, potentially compensatory hyperphagia. This paradox may reflect the complex interactions between PACAP signaling and stress-adaptive metabolic processes. In addition, previous studies by Nguyen *et al*. [17] and Jiang *et al*. [42] have shown that PACAP KO mice exhibit increased exploratory behavior and reduced stress sensitivity, possibly due to impaired PACAP signaling in limbic circuits or changes in dopaminergic tone. These observations further support the key role of PACAP in regulating both motivational behavior and the stress response.

### PAC1 receptor stability and functional implications

A previous study by Lezak *et al*. [43] has shown that PACAP and PAC1R messenger RNA (mRNA) expressions increase in the BNST following chronic, but not acute, restraint stress, correlating with elevated glucocorticoid levels. PACAP also enhances neuronal signaling in the basolateral amygdala and modulates key aspects of fear processing, including learning, memory consolidation, and extinction, through PAC1 receptor-dependent mechanisms [5, 44–48]. PAC1 expression is notably high in the locus coeruleus, a region central to orchestrating the fight-flight-freeze response and regulating peripheral metabolism under stress [49]. Despite these functional roles, PAC1 receptor expression remained stable across the day in this study, indicating that behavioral and feeding alterations are unlikely due to diurnal changes in receptor availability. Instead, they are more likely to be driven by fluctuations in PACAP levels or release. This stability suggests that PAC1 serves as a constant signaling platform that allows PACAP to exert context-dependent effects based on stress exposure or circadian timing.

### Study limitations and future directions

This study is limited by the lack of direct circadian rhythm measurements (e.g., clock gene expression, SCN activity, and locomotor rhythms), the use of only male mice, small group sizes, and the lack of direct receptor manipulation. Feeding behavior was inferred only from light/dark cycle comparisons, and temporal sampling or protein-level analysis did not support the claim of stable PAC1 expression. In future studies, the pharmacological manipulation of PACAP using selective agonists/antagonists and region-specific interventions in areas such as the BNST and amygdala is needed to dissociate emotional versus feeding circuits, which have implications relevant to stress-related eating disorders, obesity, and mood disorders in humans.

## CONCLUSION

This study demonstrates that PACAP plays a context-dependent but non-essential role in feeding regulation and stress adaptation. Specifically, PACAP overexpression in the VMH enhanced nocturnal feeding, confirming its role in the circadian modulation of appetite and energy balance. In contrast, chronic stress-induced hyperphagia occurred in both WT and PACAP KO mice, indicating that prolonged stress-driven feeding responses are independent of PACAP signaling and likely involve compensatory neuropeptide systems such as NPY, CRH, or ghrelin. Furthermore, PACAP overexpression reduced immobility in the forced swim and TSTs, supporting its involvement in stress resilience and mood regulation. Notably, PAC1 receptor expression remained stable across the diurnal cycle, suggesting that PACAP activity, rather than receptor abundance, underlies the observed behavioral and metabolic effects.

From a practical perspective, these findings highlight PACAP as a critical integrator of circadian and emotional cues that shape feeding behavior, offering potential therapeutic relevance for conditions such as stress-related eating disorders, obesity, and anxiety disorders. The ability of PACAP to influence mood and feeding simultaneously positions it as a promising target for interventions aimed at disorders that intersect metabolic and psychological domains.

The strength of this study lies in its use of complementary models, including PACAP KO mice, viral overexpression strategies, and behavioral assays, which together provide a multidimensional understanding of PACAP’s role. However, limitations include the exclusive use of male mice, relatively small sample sizes, the lack of direct measurements of circadian clock gene activity, and the absence of pharmacological receptor manipulation. These constraints limit the generalizability of findings and warrant cautious interpretation.

Future research should explore the role of PACAP in female models, integrate molecular analyses of circadian gene expression, and employ selective PACAP agonists and antagonists to dissect region-specific contributions of PACAP and PAC1 signaling. Comparative studies across different stress paradigms and dietary contexts would further clarify the interaction between emotional regulation and feeding.

In conclusion, PACAP is not indispensable for stress-induced hyperphagia but is a key modulator of circadian feeding and emotional behavior. Its dual influence on metabolic and affective processes underscores its importance in maintaining adaptive homeostasis. Expanding our understanding of PACAP signaling will provide valuable insights into the neurobiological underpinnings of stress, feeding, and mood, with translational implications for human health.

## AUTHORS’ CONTRIBUTION

TTN and HTTN: Collected the data, performed statistical analysis, and drafted the manuscript. TTN, TNM, HTTN, and TTTV: Conceived the study design and drafted and revised the manuscript. All authors have read and approved the final version of the manuscript.
